# Supplementary material for: Perpendicular Magnetic Anisotropy in Thin Films Enables Extraordinary Spin-Wave Phenomena: Anti-Larmor Precession, Negative Reflection and Refraction, Multireflection and Multirefraction
Source: ACS Appl Mater Interfaces. 2026 Feb 6;18(6):10539–51. doi: 10.1021/acsami.5c21700 (PMC13298818; doi:10.1021/acsami.5c21700)
Supplement: Supplementary file 1 [file am5c21700_si_001.pdf]

Supporting Information: Perpendicular magnetic anisotropy in thin films enables extraordinary spin-wave phenomena: anti-Larmor precession, negative reflection and refraction, multi-reflection and multi-refraction

Nikodem Leśniewski,[*,†,‡] Yuliya Dadoenkova,[¶] Florian F. L. Bentivegna,[†] and Paweł Gruszecki[*,‡]

†*CNRS, Lab-STICC, UMR 6285, Univ. Brest, Bretagne INP, 29238 Brest Cedex 3, France*

‡*Institute of Spintronics and Quantum Information, Faculty of Physics, Adam Mickiewicz University, Uniwersytetu Poznańskiego 2, 61-614 Poznań, Poland*

¶*Université Jean Monnet Saint-Etienne, CNRS, Institut d'Optique Graduate School, Laboratoire Hubert Curien, UMR 5516, 42023 Saint-Etienne, France*

E-mail: nikles@amu.edu.pl; gruszecki@amu.edu.pl

January 20, 2026

Derivation of the time dependencies of $m_x(t)$ and $m_z(t)$ for $\omega_x < 0$

Let us derive the time dependencies of $m_x(t)$ and $m_z(t)$ dynamic components of the magnetization for arbitrary initial conditions at $t = 0$ ($m_x(0)$, $m_z(0)$).

The linearized Landau-Lifshitz equation of thin-film uniformly magnetized along the y-axis, neglecting damping, can be written as:

$$\begin{aligned} \partial_t m_x &= \omega_x m_z, \\ \partial_t m_z &= -\omega_z m_x, \end{aligned} \tag{S1}$$

where

$$\omega_x = |\gamma|\mu_0 M_\mathrm{s}\left[\frac{H}{M_\mathrm{s}} + l_\mathrm{ex}^2 k^2 + (1-\xi(kd)) - Q\right], \quad \omega_z = |\gamma|\mu_0 M_\mathrm{s}\left[\frac{H}{M_\mathrm{s}} + l_\mathrm{ex}^2 k^2 + \xi(kd)\mathrm{sin}^2\theta\right],$$

where M_s is the saturation magnetization, $l_\mathrm{ex} = \sqrt{A/(\frac{1}{2}\mu_0 M_\mathrm{s}^2)}$ is the exchange length and A is the exchange constant, μ_0 is the permeability of vacuum, k is the wavevector modulus, d is the film thickness, Q is the reduced perpendicular magnetic anisotropy (PMA) constant ($Q = K_\mathrm{u}/(\frac{1}{2}\mu_0 M_\mathrm{s}^2)$ with K_u being uniaxial anisotropy constant), $\xi(x) = 1-(1-e^{-|x|})/|x|$, θ is the angle of spin wave (SW) propagation with respect to the bias magnetic field, and γ is the gyromagnetic ratio. Applying the Laplace transform to both equations yields expressions:

$$\begin{aligned} sM_x(s) - m_x(0) &= \omega_x M_z(s), \\ sM_z(s) - m_z(0) &= -\omega_z M_x(s), \end{aligned} \tag{S2}$$

where s is the complex variable, $M_x(s) = \mathcal{L}(m_x(t))$ and $M_z(s) = \mathcal{L}(m_z(t))$ represent the

Laplace transforms of $m_x(t)$ and $m_z(t)$, respectively. Solving these equation we can derive $M_z(s)$:

$$M_z(s) = \frac{sm_z(0) - \omega_z m_x(0)}{s^2 + \omega_x\omega_z}. \tag{S3}$$

When $\omega_x < 0$ (i.e., $Q > \frac{H}{M_\mathrm{s}} + l_\mathrm{ex}^2 k^2 + (1 - \xi(kd))$), the inverse Laplace transform results in the following time-domain solution for $m_z(t)$:

$$m_z(t) = m_z(0)\cosh\left(\sqrt{-\omega_x\omega_z}\,t\right) - m_x(0)\sqrt{-\frac{\omega_z}{\omega_x}}\sinh\left(\sqrt{-\omega_x\omega_z}\,t\right). \tag{S4}$$

Similarly, the time dependence of $m_x(t)$ can be derived as:

$$m_x(t) = m_x(0)\cosh\left(\sqrt{-\omega_x\omega_z}\,t\right) - m_z(0)\sqrt{-\frac{\omega_x}{\omega_z}}\sinh\left(\sqrt{-\omega_x\omega_z}\,t\right). \tag{S5}$$

This result shows that for negative values of ω_x, the amplitude of oscillations in the magnetization components increases over time. This growth indicates that the system becomes unstable, leading to a phase transition. However, as m_x and m_z grow, the linear approximation assuming that $m_x, m_z \ll M_\mathrm{s}$ eventually ceases to be valid. Consequently, this model can only describe the initial stages of the phase transition before nonlinear effects dominate.

Impact of damping on dispersion relation

The complete Landau-Lifshitz-Gilbert equation with damping reads:

$$\partial_t \mathbf{M} = -|\gamma|\mu_0 \mathbf{M} \times \mathbf{H}_\mathrm{eff} + \frac{\alpha}{M_\mathrm{s}} \mathbf{M} \times \partial_t \mathbf{M} \tag{S6}$$

where α is the Gilbert damping parameter and $\mathbf{H}_\mathrm{eff} = \mathbf{H} + \mathbf{H}^\mathrm{ex} + \mathbf{H}^\mathrm{d} + \mathbf{H}^\mathrm{PMA}$ represents the effective magnetic field. This field includes the external field $\mathbf{H} = H\hat{\mathbf{e}}_y$, exchange field $\mathbf{H}^\mathrm{ex} = l_\mathrm{ex}^2 M_\mathrm{s} \nabla^2 \mathbf{M}$, dipolar field $\mathbf{H}^\mathrm{d}$, and perpendicular magnetic anisotropy field $\mathbf{H}^\mathrm{PMA} =$

$Qm_z\hat{\mathbf{e}}_z$, respectively, where $\hat{\mathbf{e}}_y$ and $\hat{\mathbf{e}}_z$ are unit vectors along the y and z directions.

Following the linearization procedure around the equilibrium magnetization state, the linearized Landau-Lifshitz-Gilbert equation yields:

$$\partial_t m_x = -|\gamma|\mu_0 M_\mathrm{s}\left(-\frac{H}{M_\mathrm{s}} + l_\mathrm{ex}^2\nabla^2 m_z + h_z^\mathrm{d} + Qm_z\right) + \alpha\partial_t m_z \tag{S7}$$

$$\partial_t m_z = |\gamma|\mu_0 M_\mathrm{s}\left(-\frac{H}{M_\mathrm{s}} + l_\mathrm{ex}^2\nabla^2 m_x + h_x^\mathrm{d}\right) - \alpha\partial_t m_x \tag{S8}$$

For an ultra-thin layer, the dipolar field can be written as $\mathbf{h}^\mathrm{d} = \left[h_x^\mathrm{d}, 0, h_z^\mathrm{d}\right] =$ $= \left[-\xi(kd)\sin^2(\theta)m_x,\ 0,\ -(1-\xi(kd))m_z\right]$. Assuming plane wave solutions of the form $m_{x,z} \propto e^{i(kx-\omega t)}$, this yields:

$$\begin{aligned} -i\omega m_x &= \omega_x m_z - i\omega\alpha m_z \\ -i\omega m_z &= -\omega_z m_x + i\omega\alpha m_x \end{aligned} \tag{S9}$$

Solving this system of equations leads to the following quadratic equation:

$$(1+\alpha^2)\omega^2 + i\alpha(\omega_x + \omega_z)\omega - \omega_x\omega_z = 0 \tag{S10}$$

The solution of this quadratic equation for $\mathrm{Re}(\omega) > 0$ is:

$$\omega = \frac{\sqrt{\omega_0^2 - \frac{\alpha^2}{4}(\omega_x - \omega_z)^2}}{1+\alpha^2} - i\frac{\alpha}{2(1+\alpha^2)}(\omega_x + \omega_z), \quad \omega_0 = \sqrt{\omega_x\omega_z} \tag{S11}$$

The analysis of eq. S11 reveals four key features:

1. *Undamped limit*: When $\alpha = 0$, the solution reduces to $\omega = \omega_0$, which recovers the dispersion relation derived in the main manuscript.

2. *Finite linewidth*: The imaginary part of ω introduces exponential decay, directly related

to the linewidth observed experimentally.

3. *Damping-induced frequency shift*: The real part of ω is modified by the damping parameter, leading to a shift in the frequency that scales quadratically with α. To obtain a closed-form approximation for $\mathrm{Re}\,\omega$, we perform a small-damping perturbative expansion—namely, a Taylor (binomial) series in α about $\alpha = 0$—retaining terms up to $O(\alpha^2)$:

$$\mathrm{Re}(\omega) \approx \omega_0 - \alpha^2 \left[\omega_0 + \delta\right], \quad \delta = \frac{(\omega_x - \omega_z)^2}{8\omega_0} \tag{S12}$$

Therefore, the approximated damping-induced frequency shift of the dispersion is:

$$\Delta\omega = \mathrm{Re}(\omega) - \omega_0 = -\alpha^2 \left[\omega_0 + \delta\right]. \tag{S13}$$

This expression reveals that the frequency shift scales quadratically with the damping parameter α and always reduces the resonance frequency below its undamped value $\omega_0 = \sqrt{\omega_x\omega_z}$. In the limit where $|\omega_z - \omega_x| \ll 8\omega_0$, we recover the well-known result from the literature:[1] $\Delta\omega \approx -\alpha^2\omega_0$, which corresponds to the standard approximation $\omega_0/(1+\alpha^2) \approx (1-\alpha^2)\omega_0$ for circular precession.

However, when the precession becomes elliptical (i.e., $\omega_x \neq \omega_z$), a second contribution emerges: $-\alpha^2(\omega_x - \omega_z)^2/(8\omega_0)$. This ellipticity-induced correction becomes particularly significant when $\omega_0 \to 0$, which occurs as $\omega_x \to 0$. In this limit, the frequency shift is dominated by the ellipticity term, leading to $\Delta\omega \approx -\frac{\alpha^2\omega_z^2}{8\omega_0} = -\frac{\alpha^2\omega_z^{3/2}}{8\sqrt{\omega_x}}$, which diverges as $\omega_x \to 0$, fundamentally altering the damping behavior in systems with highly elliptical precession.

4. *Critical behavior*: Analysis of not approximated equation for the dispersion with damping Eq. (S11) reveals that when either ω_x or ω_z vanishes (and therefore $\omega_0 = 0$), the

frequency shift $\Delta\omega$ also vanishes, indicating that the damping-induced correction disappears exactly when the undamped system reaches zero frequency.

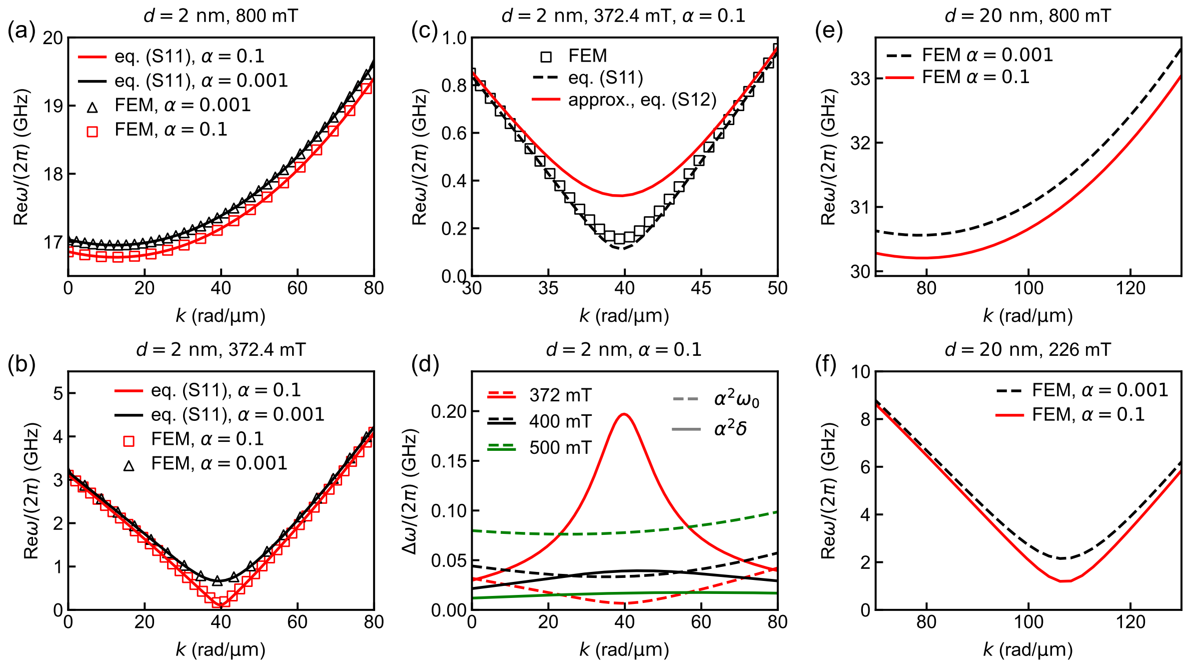

Fig. S1: Damping–induced dispersion and scaling in CoFeB (M_s = 1344 kA/m, A_ex = 13.6 pJ/m, Q = 1.2, γ = 176 GHz/T). We plot the frequency $f = \mathrm{Re}\,\omega/(2\pi)$ versus wave number k (rad/μm). Panels (a)–(c) address the ultrathin film (d = 2 nm): in (a) for $\mu_0 H$ = 800 mT and in (b) for $\mu_0 H$ = 372.4 mT, the solid red and black curves show the analytical model of Eq. (S11) for $\alpha = 0.1$ and $\alpha = 0.001$, respectively, while symbols denote FEM results; panel (c) focuses on $\mu_0 H$ = 372.4 mT and compares FEM (symbols) with the exact analytical expression (black dashed line, Eq. (S11)) and its approximation (red solid line, Eq. (S12)). Panel (d) presents the frequency shift introduced by the damping for $d = 2$ nm and $\alpha = 0.1$ by plotting $\alpha^2\omega_0$ (solid lines) and $\alpha^2\delta$ (dashed lines) versus k for $\mu_0 H = 372.4$, 400, and 500 mT; color encodes the field (red, black, green), line style encodes the quantity. Panels (e)–(f) show the thin film (d = 20 nm) at $\mu_0 H \approx 226$ mT: high–k and low–frequency ranges, respectively, with FEM dispersions for $\alpha = 0.1$ (red solid) and $\alpha = 0.001$ (black dashed).

Figure S1 quantifies how Gilbert damping reshapes the spin–wave dispersion and validates the analytical model in Eq. (S11). Figures S1(a–d) address the ultrathin film ($d = 2$ nm), while Figures S1(e–f) show a 20-nm CoFeB film (materials parameters as in the caption).

For the ultrathin film, Figures S1(a) and (b) demonstrate excellent quantitative agreement between the exact analytical dispersion (Eq. S11, solid curves for $\alpha = 0.1$ in red

and $\alpha = 0.001$ in black) and finite-element (FEM) solutions of the linearized Landau-Lifshitz-Gilbert equation and Finite Element-Method (FEM) simulations solving numerically linearized Landau-Lifshitz-Gilbert equation (S7) (symbols; see Methods). This one-to-one match across fields $\mu_0 H = 800$ mT and 372.4 mT confirms the correctness of the closed-form model.

Figure S1(c) compares the small-α approximation (Eq. S12, red line) against the exact formula (black dashed) and FEM near the softening regime ($\mu_0 H = 372.4$ mT). The approximation tracks the data over most of k, but underestimates the downward shift where the mode approaches its minimum and the ellipticity term δ becomes non-negligible—consistent with the stated scope of validity (it degrades as $\omega_0 \to 0$). To improve agreement in this regime, the series should be extended beyond $O(\alpha^2)$, i.e., by retaining higher–order terms.

Figure S1(d) separates the quadratic damping shift, $\Delta\omega \simeq -\alpha^2(\omega_0 + \delta)$, into its two components by plotting $\alpha^2\omega_0$ (gray dashed) and $\alpha^2\delta$ (gray solid) for three fields. The total shift (colored curves) is maximized when the undamped frequency is small, and the precession is strongly elliptical (large $(\omega_x - \omega_z)^2$). In particular, at $\mu_0 H = 372$ mT the $\alpha^2\delta$ contribution dominates, whereas its relative weight decreases monotonically with field and is already much smaller than $\alpha^2\omega_0$ at 400 mT. Thus, the model predicts that the damping– and ellipticity–induced frequency reduction should be experimentally resolvable under appropriate conditions at the fields approaching the critical value and for wave vectors at which the mode frequency tends toward zero.

For the 20-nm film, panels (e) and (f) show the same qualitative behavior in FEM: damping systematically lowers $\mathrm{Re}\,\omega$, with a stronger relative effect at lower fields ($\mu_0 H \approx 226$ mT) where the dispersion has a deeper minimum.

Overall, the simulations reproduce the analytical predictions panel-by-panel: (i) the frequency shift is negative and scales $\propto \alpha^2$, (ii) it is maximized near softening, where δ dominates, and (iii) the small-α approximation accurately captures the trend away from the soft-mode limit. Moreover, for systems with damping favorable to spin-wave propagation

($\alpha \lesssim 10^{-2}$), the damping-induced modification of the dispersion is negligible across most of k and H; detectable effects arise primarily as the field approaches the critical value and for wave vectors where $\mathrm{Re}\,\omega \to 0$, i.e., precisely when the ellipticity term δ becomes significant.

To quantify the impact of damping on the IFCs, we have calculated the contours for different values of the Gilbert damping parameter ($\alpha = 10^{-3}$, and 10^{-2}) and for various external magnetic field strengths and frequencies. The results shown in Fig. S2 clearly demonstrate that for damping values $\alpha \leq 10^{-2}$, the differences in the isofrequency contours are negligible.

Torques

We analyze the behavior of the mode profile and torque components as functions of the z-coordinate (across the film thickness) for three values of the wavevector modulus: $k = 46$, 106, and 186 rad/µm, in a 20-nm-thick film subjected to an external magnetic field of 250 mT. The analyzed parameters are extracted from the first dispersion branch shown in Figure 2(a) of the main text, where they are marked by red dots labeled (h), (d), and (j). Figures S3(a)-(c) show the spatial distributions of m_x and m_z at the same time. Figures S3(d)-(f) and S3(g)-(i) depict the contributions of the x- and y-components of the effective torque τ_x^{eff} and τ_y^{eff}, respectively, acting on the magnetization. These torque contributions are computed using Eq. (2) from the main text and normalized for comparison. For all cases, τ_z^{eff} does not change sign. For $k = 106$ rad/µm, corresponding to the point in the spectrum where anti-Larmor precession occurs, both m_x and the overall x-component of the torque, τ_x^{eff}, cross zero. In contrast, for $k = 46$ rad/µm and $k = 186$ rad/µm, neither m_x nor τ_x^{eff} cross zero.

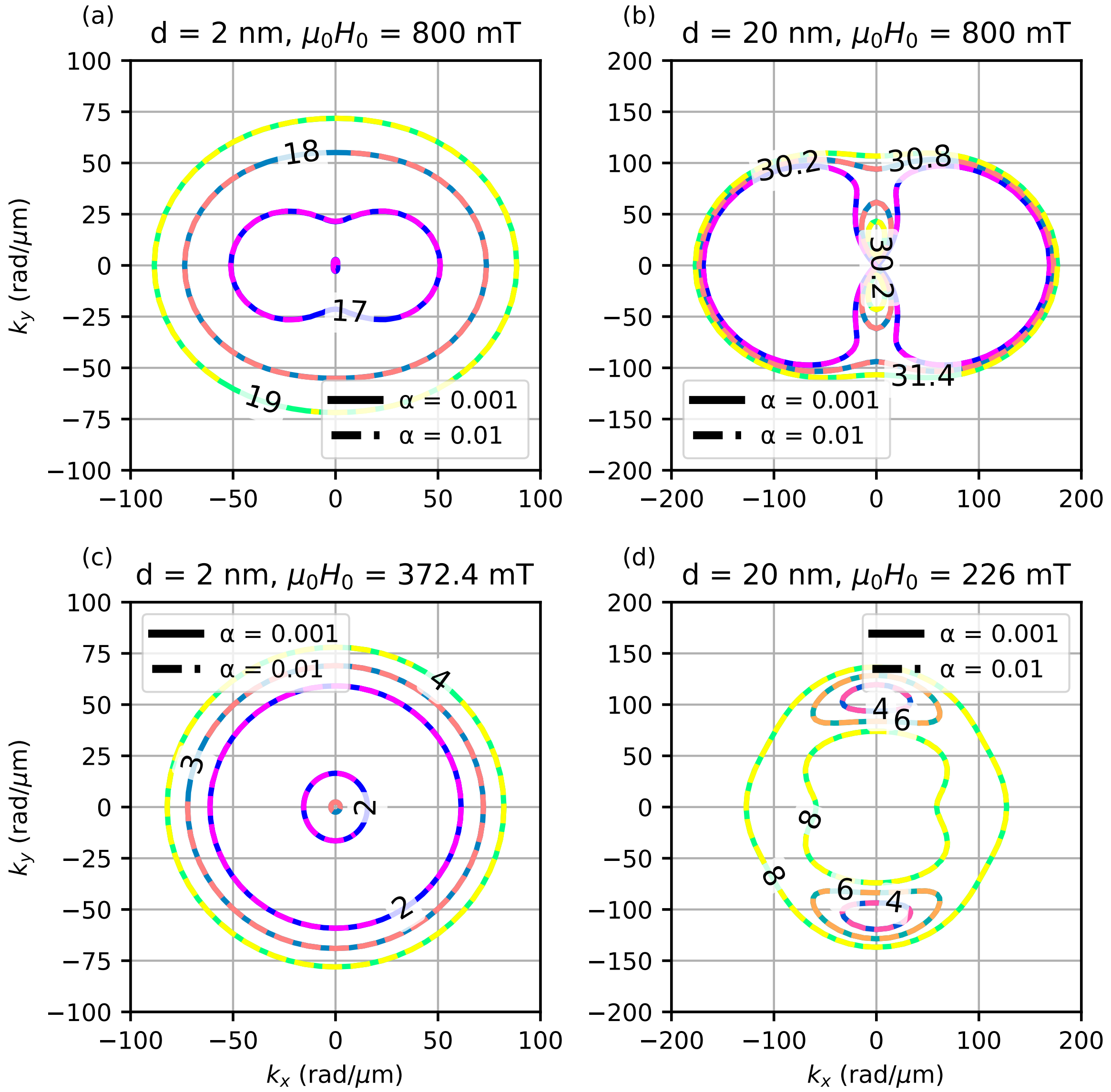

Fig. S2: IFCs in CoFeB ($M_s = 1344$ kA/m, $A_{ex} = 13.6$ pJ/m, $\gamma = 176$ GHz/T) with different damping constants. We plot the IFCs in ultrathin film (2 nm, $Q = 1.2$) in the external field (a) 800 mT and (c) 372.4 mT, and in thin film (20 nm, $Q = 0.6$) in (b) 800 mT and (d) 226 mT. The solid lines correspond to a damping constant $\alpha = 0.001$ and the dashed lines to $\alpha = 0.01$. Different line colors correspond to frequency values for which the contours were extracted. The frequency values are indicated inline in GHz.

Interference of spin waves

The shape of the dispersion relation with a minimum at nonzero k and the precession of magnetic moments associated with the bottom of this relation for spin waves propagating

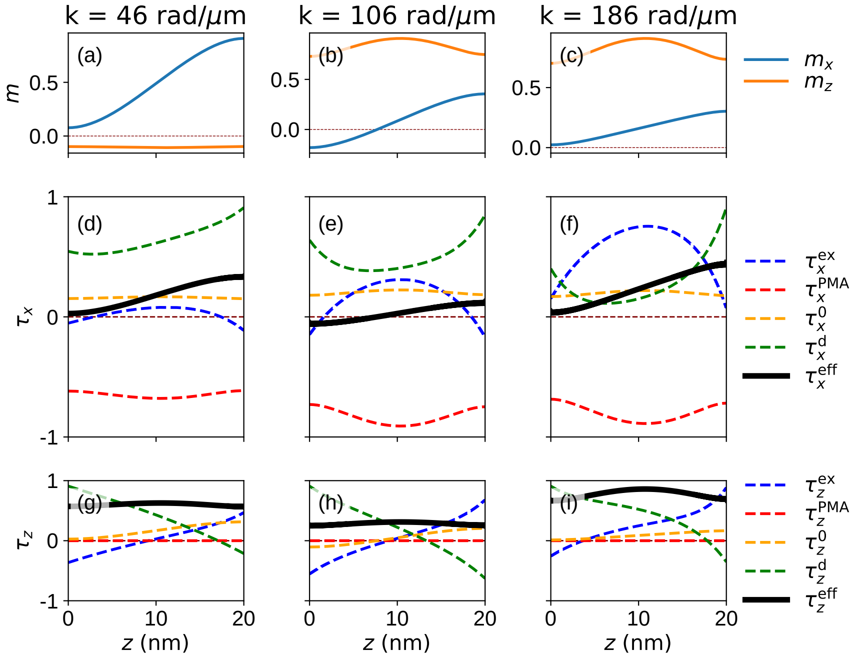

Fig. S3: Dependencies over the z-coordinate are shown for (a)-(c) the mode profiles of spin waves (SWs) (m_x—blue line, and m_z—orange line); (d)-(f) the x-component and (g)-(i) the z-component of the torque contributions acting on the magnetization. The results are presented for three different values of the wavevector modulus k corresponding to the red dots (h), (d), and (j) in Fig. 2(a) of the main text: 46 rad/µm (left column), 106 rad/µm (central column), and 186 rad/µm (right column). (d)-(g) The dashed blue, green, red, and orange lines represent the exchange, dipolar, PMA, and Zeeman contributions to τ, respectively, with the solid black line denoting the effective torque.

perpendicular to the applied field can lead to intriguing effects.

Consider a 20-nm-thick CoFeB layer magnetized by a 250 mT magnetic field (the same system as in the main part of the work). The dispersion relation for this system is shown in Fig. S4(d). Excitation with a conventional microwave antenna generates spin waves with different wave vectors (with efficiency depending on the antenna geometry). For simplicity, we assume that the dynamic magnetic field has the form $b_z \propto \exp[-2.77(x/w)^2]$, where $w = 25$ nm is the full width at half maximum of the Gaussian distribution. Such a field effectively excites spin waves with wavelengths larger than 50 nm.

Micromagnetic simulation results for excitation with an 8 GHz field are presented in Fig. S4. Panel (a) shows precession orbits at different positions along the x and y axes. In certain regions (top for left-propagating waves, bottom for right-propagating waves), the

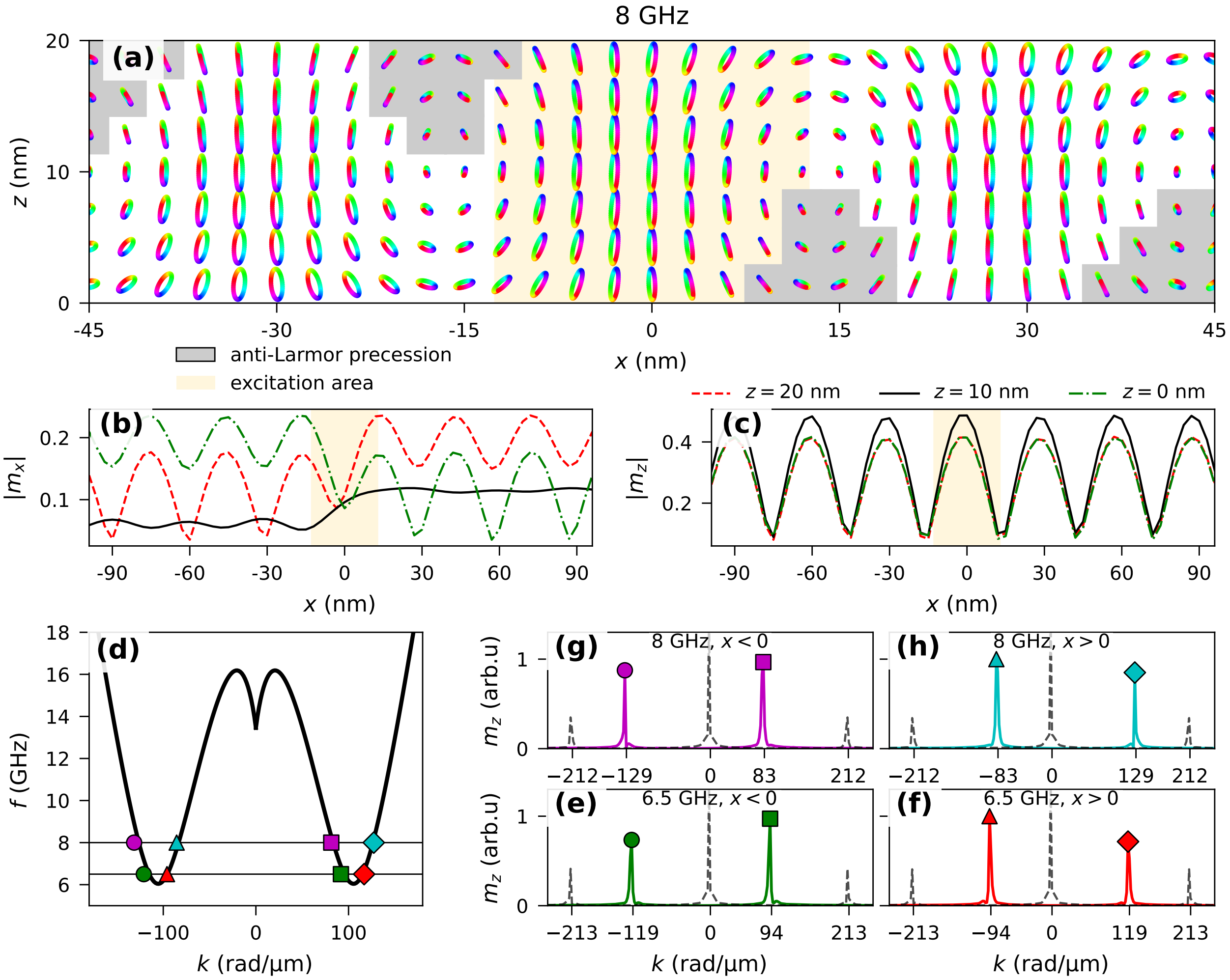

Fig. S4: Interference of spin wave modes with opposite phase velocities in a 20-nm-thick CoFeB layer magnetized by a 250 mT in-plane magnetic field. (a) Precession orbits at different spatial positions for 8 GHz excitation. Colors along each orbit indicate the temporal evolution of magnetic moment orientation (as in the main text). Gray shaded regions mark areas where anti-Larmor precession occurs. Yellow shaded areas in (a-c) indicate the Gaussian excitation region with $w = 25$ nm width. (b,c) Spatial distribution of $|m_x|$ and $|m_z|$ showing oscillating amplitude due to interference of counter-propagating modes. (d) Dispersion relation with marked points corresponding to excited wave numbers at 8 GHz (the same symbols as in (g-h)) and 6.5 GHz (the same symbols as in (e-f)). (g,h) Spatial Fourier transform of m_z for $x < 0$ (left propagation) and $x > 0$ (right propagation) at 8 GHz, revealing mode pairs with opposite k but same sign of group velocity. Narrow black dashed lines represent Fourier transform of $|m_z|$ showing peaks at $k = 0$ and $k = \pm 212$ rad/μm corresponding to the interference pattern period. (e,f) Analogous results to (b,c) for 6.5 GHz excitation.

precession direction changes to anti-Larmor. It is important to note that for a single spin wave mode, as shown in the main part of the work, the precession orbits and their hand-

edness are independent of position x. However, in experiments with broadband excitation, the interference of two simultaneously excited modes leads to periodic spatial variations of precession orbits along the x axis, including changes in the precession direction. The directional change is visible only in certain areas because the image results from interference of waves with opposite signs of phase velocity but identical directions of group velocity.

This results in real-space interference, visible in Fig. S4(b-c), which shows the absolute value of complex-valued magnetization $|m_x|$ and $|m_z|$. The amplitude oscillates even though excitation occurred around $x \approx 0$. The Fourier transform of the m_z distribution, calculated separately for $x < 0$ and $x > 0$ (Fig. S4(g) and (h)), reveals peaks at $k = -129$, 83 rad/μm (left propagation) and $k = -83$, 129 rad/μm (right propagation).

These wave numbers correspond to $f = 8$ GHz on the dispersion curve in Fig. S4(d). For right propagation, both solutions ($k = -83$ and 129 rad/μm) have $v_g > 0$ despite opposite phase velocity directions, causing interference. The situation for left propagation is analogous.

Confirmation of this interpretation is provided by results for $f = 6.5$ GHz (Fig. S4(e-f)), with corresponding points overlaid on Fig. S4(d). The FFT of $|m_z|$ (plots (g)-(i)) shows additional peaks at $k = 0$ and $k = \pm 212$ rad/μm for $f = 8$ GHz ($k = \pm 213$ rad/μm for $f = 6.5$ GHz). The value $k = 212$ rad/μm $= 129 + 83$ rad/μm corresponds to the spatial period of the interference pattern.

In conclusion, the unique topology of the dispersion relation with a minimum at finite k leads to simultaneous excitation of mode pairs with k and $-k'$ having the same sign of v_g. The resulting interference fundamentally alters the spatial distribution of magnetization dynamics compared to single-mode propagation. This has direct implications for experimental studies: (i) spatially resolved measurements will exhibit position-dependent apparent precession characteristics, (ii) standard broadband excitation schemes cannot selectively address individual modes, and (iii) quantitative analysis of spin wave transport requires either narrowband excitation near a single k value or explicit modeling of multi-mode interference.

These considerations are essential for accurate characterization and utilization of spin wave systems with non-monotonic dispersion.

Method comparison

In Fig. S5, we show the comparison of two methods for calculating the dispersion relation. The agreement between mumax3 and COMSOL Multiphysics calculation is visible for high (top panels) and low (bottom panels) fields, as well as for ultrathin (left panels) and thin (right panels) films.

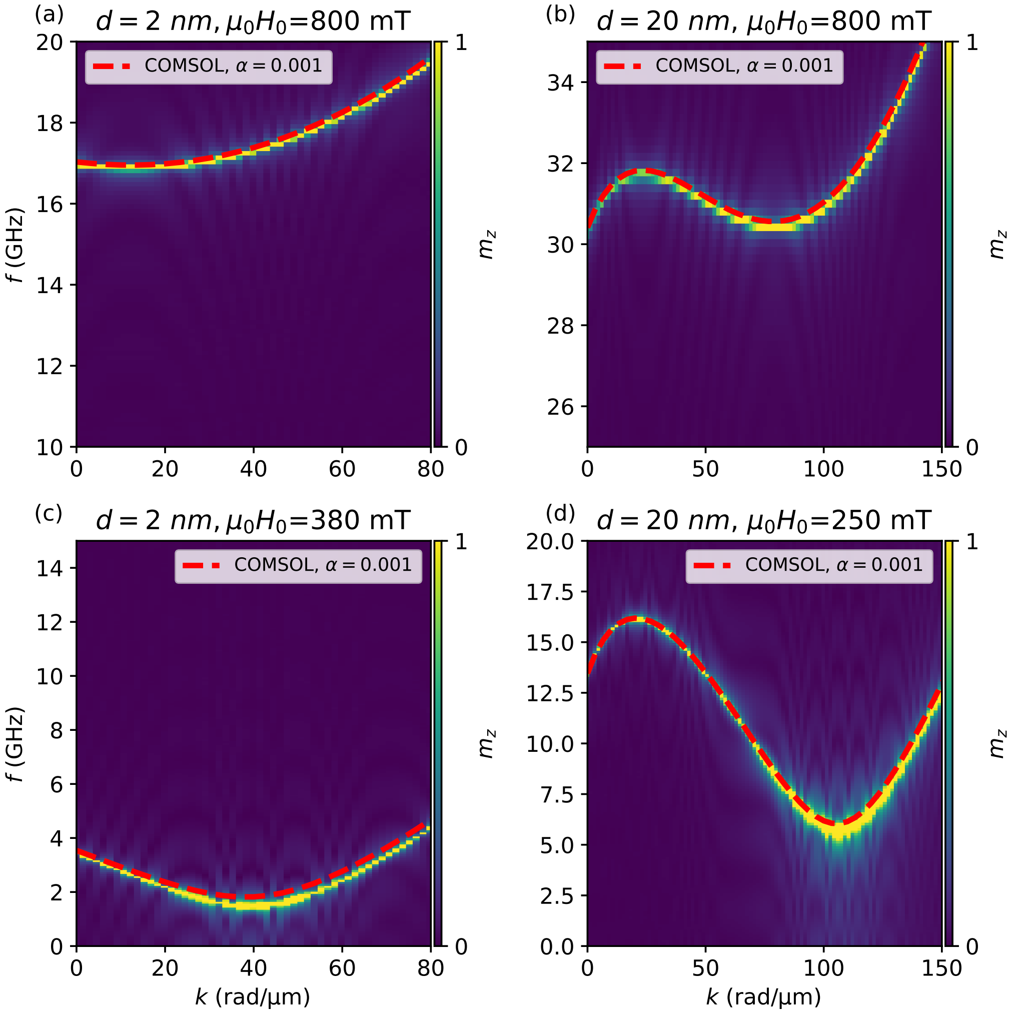

Fig. S5: The dispersion relations in CoFeB ($M_s = 1344$ kA/m, $A_{ex} = 13.6$ pJ/m, $\gamma = 176$ GHz/T) calculated using two methods. The colormap corresponds to the dispersion calculated from the normalized m_z component of magnetization obtained from mumax3 calculations, and the dashed line was obtained from COMSOL. We plotted the dispersion relations for ultrathin film (a, c) (2 nm, $Q = 1.2$), and thin film (b, d) (20 nm, $Q = 0.6$) in external field values (a) 800 mT and (c) 380 mT for ultrathin film and (b) 800 mT and (d) 250 mT for thin film

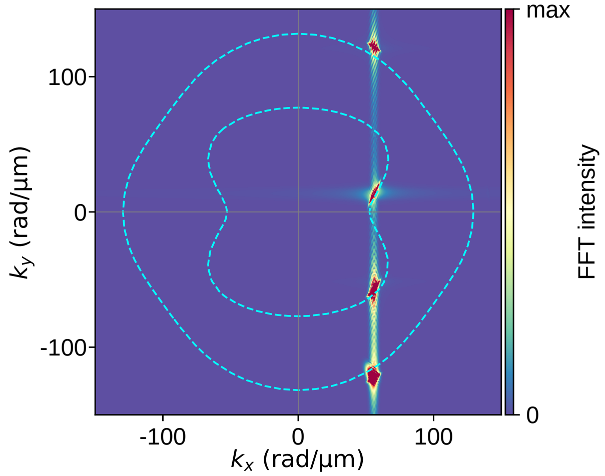

Fig. S6: Large-scale version of of the results shown in Fig. 3(b) of the main manuscript. The colormap in the background represents the two-dimensional FFT of the result of micromagnetic simulations shown in Fig. 3(c). The dashed cyan line represents the IFC computed employing COMSOL Multiphysics.

References

(1) Gurevich, A. G.; Melkov, G. A. *Magnetization Oscillations and Waves*; CRC Press: Boca Raton, 1996.
